# Rap1 and Cdc13 have complementary roles in preventing exonucleolytic degradation of telomere 5′ ends

**DOI:** 10.1038/s41598-017-08663-x

**Published:** 2017-08-18

**Authors:** Rikard Runnberg, Saishyam Narayanan, Marita Cohn

**Affiliations:** Department of Biology, Genetics group, Lund University, Lund, Sweden

## Abstract

Telomere DNA ends with a single-stranded 3′ overhang. Long 3′ overhangs may cause aberrant DNA damage responses and accelerate telomere attrition, which is associated with cancer and aging, respectively. Genetic studies have indicated several important players in preventing 5′ end hyper-resection, yet detailed knowledge about the molecular mechanism in which they act is still lacking. Here, we use an *in vitro* DNA 5′ end protection assay, to study how *N. castellii* Cdc13 and Rap1 protect against 5′ exonucleolytic degradation by λ-exonuclease. The homogeneous telomeric repeat sequence of *N. castellii* allows us to study their protection ability at exact binding sites relative to the 5′ end. We find efficient protection by both Cdc13 and Rap1 when bound close to the 5′ end. Notably, Rap1 provides protection when binding dsDNA at a distance from the 5′ end. The DNA binding domain of Rap1 is sufficient for 5′ end protection, and its wrapping loop region is essential. Intriguingly, Rap1 facilitates protection also when its binding site contains 2 nt of ssDNA, thus spanning across the ds-ss junction. These results highlight a role of Rap1 in 5′ end protection and indicate that Cdc13 and Rap1 have complementary roles in maintaining proper 3′ overhang length.

## Introduction

Telomeres are the nucleoprotein structures that protect the end of eukaryotic chromosomes. The cell must distinguish telomeres from double stranded (ds) DNA breaks to prevent aberrant DNA damage response (DDR), leading to genomic instability, cell death or senescence. Telomere DNA consists of repetitive G-rich sequence (referred to here as G-strand), ending in a single-stranded 3′ overhang, and a complementary 5′ resected C-rich strand (referred to here as C-strand). The conventional replication machinery is unable to replicate the very end of chromosomes, leading to the requirement of additional machineries for carrying out telomere length maintenance^1^. In most eukaryotes telomere length is maintained by telomerase, a reverse transcriptase that carries its own RNA template and adds telomere repeats to the terminal 3′ overhang^2–5^. The length of telomere 3′ overhangs varies considerably during the cell cycle and needs to be regulated for maintaining telomere integrity^6, 7^. Intriguingly, the 3′ overhang length is proportional to the rate of telomere shortening in telomerase deficient cell lines, meaning cells with longer overhangs have a faster rate of telomere shortening^8^. Since most cells of human tissues are telomerase deficient, it was proposed that 3′ overhang length may regulate the rate of telomere shortening and accompanying cellular senescence^8^. Hence, regulation of telomere overhang length may be important both for avoiding genetic instability and cancer, as well as for controlling the rate of normal aging.

In human cells the length of the 3′ overhang has a wide distribution from only a few up to several hundred nucleotides, being lengthened from an average of ~65 nt to ~105 nt during S-phase in HeLa cells^9^. This overhang lengthening is carried out by telomerase, occurring shortly after replication by the conventional replication machinery, and is then followed by C-strand fill-in by a separate machinery that concomitantly reduces overhang length^9^. The occurrence of the same 5′ end permutation on nearly every human telomere strongly suggests that the generation of the 3′ overhang is a precisely regulated mechanism^10^. The budding yeast *Saccharomyces cerevisiae* maintains a telomere 3′ overhang length of 12–14 nt for most of the cell cycle, only transiently lengthening it to >30 nt in S-phase^7, 11^. In *S. cerevisiae* both telomere replication by the conventional replication machinery and lengthening by telomerase occur in late S/G2 phase and the longer overhangs detected in S-phase are presumably created by 5′ end resection rather than telomerase^12–14^. Leading strand replication will leave a blunt end unsuitable for lengthening by telomerase, hence 5′ end resection is hypothesised to occur to allow lengthening by telomerase^15^. Indeed, 5′ end resection occurs also on mammalian telomeres, where the 5′ exonuclease Apollo is recruited to the leading strand, while Exo1 acts on both leading and lagging strand telomeres^16–18^. Also in *S. cerevisiae*, Exo1 has a role in 5′ end resection, although other 5′ exonucleases that remain to be characterized are also known to act on the telomere^19^. The Mre11-Rad50-Xrs2 (MRX) complex is important for maintaining the constitutive *S. cerevisiae* 10–14 nt telomere 3′ overhangs, and together with helicases Sae2 and Sgs1 it allows nucleases Exo1 and DNA2 access to de novo telomeres^11, 20^.

Several proteins that bind specifically to telomere DNA to regulate telomere length and prevent aberrant DDR have been identified. In *S. cerevisiae* Cdc13 is an essential protein that binds sequence-specifically to the G-strand 3′ overhang^21^. Cdc13 has a key function to recruit telomerase to the 3′ end by direct interaction with the Est1 component of the telomerase holoenzyme^22, 23^. In addition, it is a part of a complex together with Stn1 and Ten1, the CST complex, which has multiple roles in telomere maintenance^24, 25^. Mutant strains carrying the temperature sensitive *Cdc13-1* allele experience hyper resection of the 5′ end by Exo1, resulting in long overhangs that are recognized by the DDR^6, 21^. This indicates a role for Cdc13 in protection from 5′ resection, and it was recently shown that Cdc13 indeed inhibits resection by λ-exonuclease in a biochemical assay^26^. However, Cdc13 was found to be dispensable for telomere protection in non-dividing cells and was therefore proposed to perform a specialized protection within just a narrow time window late in S phase^27^. The major telomere protein binding to dsDNA in *S. cerevisiae* is Rap1, which is a key regulator of telomere length and stability^28, 29^. Rap1 binds telomere dsDNA via a DNA binding domain (DBD) containing two Myb sub-domains^30^. Recently, a particular region of Rap1, the so-called wrapping loop, was found to play a role in locking the two Myb domains in place on DNA, and was shown to be essential for Rap1 function *in vivo* ^31^. In conjunction with its interacting partners Rif1 and Rif2, Rap1 is a negative regulator of telomerase elongation^32, 33^. In contrast to Cdc13, Rap1 was found to be crucial for preventing accumulation of single stranded (ss)DNA in both non-dividing and cycling cells^27^. It was proposed that cell-cycle-specific processing may occur, where Rap1 would act in preventing 5′ end resection in G0/G1-phase, while Cdc13 would be crucial for preventing excessive 5′ end resection in S-phase^27^.

In mammals the core protein complex at telomeres is the six-membered Shelterin complex^34^. The most highly conserved protein in the Shelterin complex is Rap1, although rather than interacting directly with telomere dsDNA (as in *S. cerevisiae*) it binds telomeres primarily via the Shelterin protein TRF2^35^. Although many functions of yeast Rap1 appears to be harnessed by other Shelterin members in mammalian cells, a recent report showed that mammalian Rap1 is needed for preventing telomere 5′ end resection and homology directed repair (HDR) when the basic domain of TRF2 has been deleted^36^. Human homologs of the CST complex have a role in regulating 3′ telomere overhang length, albeit primarily by regulating a C-strand fill-in mechanism rather than by inhibiting Exo1^16^.

The budding yeast *Naumovozyma castellii* (also known as *S. castellii*), which belongs to the family of Saccharomycetaceae, has been extensively used for studies in comparative genomics and molecular evolution. Its amenability for genetic modification has rendered it a feasible model organism for molecular genetic functional analyses and it has significantly contributed with discoveries of fundamental molecular mechanisms in the fields of centromeres, mitochondria, RNAi and telomeres^37^. The telomeres of *N. castellii* share many features with those of human cells. They contain homogeneous 5′-TCTGGGTG-3′ repeats and recent investigations by our group revealed that their telomere 3′ overhangs are highly variable in length, ranging from about 14–200 nt in unsynchronized cells, while accumulating 70 nt overhangs in S-phase^38, 39^. We previously characterized the telomere-specific DNA binding activities of the *N. castellii* Cdc13 and Rap1 homologs^40, 41^. Cdc13 requires a minimum of eight specific nucleotides of telomere ssDNA for its high affinity binding (5′-GTGTCTGG-3′), defined as its minimal binding site (MBS)^40^. Rap1 binds specifically to a 12 bp MBS (5′-GGGTGTCTGGGT-3′) on telomere dsDNA^41, 42^. The Rap1 MBS contains two half-sites (5′-GGTGT-3′ and 5′-TGGGT-3′) separated by one base redundant for high-affinity binding, where each half-site presumably represents the binding sites of each Rap1 Myb-subdomain^30, 42^. Nevertheless, the sequence specificities of Cdc13 and Rap1 to the telomeric sequence show a significant degree of overlap, and recently *N. castellii* Rap1 was found not only to bind its canonical dsDNA binding site, but also to bind across the ds-ss junction, where it can compete with Cdc13 binding at certain 5′ end permutations^42, 43^.

While genetic studies in yeast and mammalian cells have revealed many of the important players in 5′ end protection, detailed knowledge about the molecular mechanism in which they act is still lacking. In this work we take advantage of the regular repeat structure of the *N. castellii* telomere sequence, to study how telomere binding proteins may protect from 5′ end resection. We employ an *in vitro* λ-exonuclease based DNA 5′ end protection assay (DEPA) to study whether binding of Cdc13 versus Rap1 to *N. castellii* telomere ends may protect from 5′ exonucleolytic degradation. By using DNA substrates with various different 5′ end permutations, and hence varying distances between the Cdc13 and Rap1 binding sites to the ds-ss junction, we study the positional effect of Cdc13 and Rap1 binding on the protection from λ-exonuclease degradation. We find that Cdc13 and Rap1 have complementary roles in 5′ end protection, both being able to protect the 5′ end from exonucleolytic degradation when bound immediately adjacent to the ds-ss junction and further away on the ssDNA and dsDNA, respectively. Notably, this indicates that Rap1 will be able to protect the 5′ end when the 3′ overhang is too short for Cdc13 to bind. We also show that the Rap1 DBD is sufficient for mediating 5′ end protection, and that the wrapping loop of the DBD is essential for this function.

## Results

### *N. castellii* Cdc13 protects the telomere 5′ end from exonucleolytic degradation

**Figure 1:**
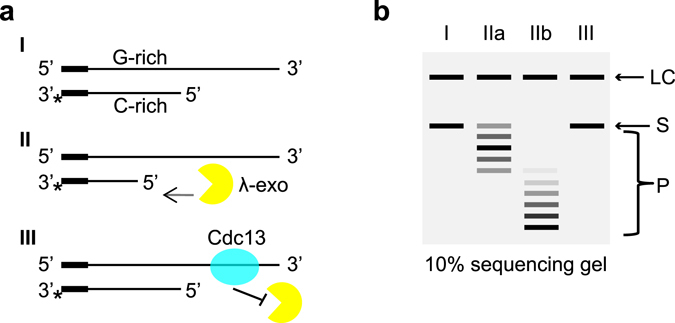
(**a**) Schematic illustration of the 5′ DNA end protection assay (DEPA). DNA oligonucleotides are annealed to form model telomeres with a double stranded part and a single stranded 3′ overhang (I). All oligonucleotides contain a short non-telomeric guide sequence to ensure efficient annealing while the telomere part is varied to create different length overhangs and different 5′ permutations. λ-exonuclease selectively cleaves the 5′ phosphorylated end (II) of the shorter C-strand oligonucleotide which is 3′ end labelled (*). The reaction progresses in the 5′ → 3′ direction (II). To assay for 5′ end protection, Cdc13 is pre-bound to the telomere end before adding λ-exonuclease to the reaction, which will inhibit the exonuclease (III). (**b**) Schematic illustration of the assay read out. Reactions are stopped at different incubation times, de-proteinized, ethanol precipitated and run on a 10% denaturing polyacrylamide sequencing gel. A labelled oligonucleotide loading control (LC) is added before ethanol precipitation which migrates above the 3′ labelled C-strand substrate (S) on the gel. As the exonuclease reaction progresses, products of decreasing size (P) appears on the gel while the uncleaved substrate (S) diminishes. Lane I, no enzyme control (0 s); lane IIa, shorter incubation time; lane IIb, longer incubation time; lane III, a reaction where the substrate was pre-incubated with Cdc13 which gave full protection.

To gain insight of how Cdc13 binding to the 3′ telomere overhang interferes with 5′ end exonucleolytic degradation we developed a λ-exonuclease based DNA end protection assay (DEPA). λ-exonuclease was chosen as a commercially available substitute to yeast Exo1, which is difficult to obtain in large enough quantities as recombinant protein, because it has similar characteristics as Exo1 in terms of processivity and has been successfully used for telomere protection studies^26^. We focused our studies on the budding yeast *N. castellii*, which has homogeneous 8 bp telomere repeats. Hence the position of the first Cdc13 binding site on the 3′ overhang in relation to the ds-ss junction will be determined by the 5′ end permutations, and thus the positional effect of Cdc13 binding relative to the ds-ss junction can be studied. The oligonucleotides used in DEPA contain the *N. castellii* telomere repeat sequence and a short non-telomeric guide sequence. They are annealed to produce substrates with blunt ended non-telomeric sequence at one end and telomere DNA with protruding 3′ overhangs at the other end, thus mimicking the *N. castellii* telomere (panel I, Fig. 1a). The C-strand is phosphorylated at its 5′ end to be selectively targeted by λ-exonuclease and is 3′ radiolabelled for its visualization on the sequencing gel (panel II, Fig. 1a). The exonuclease gradually degrades the C- strand until the reaction is stopped. To assess the protection by Cdc13, the telomere substrate is pre-incubated with Cdc13 protein before the λ-exonuclease is added (panel III, Fig. 1a). The reaction products are resolved on a sequencing gel (Fig. 1b). If protected, the C- strand will remain uncleaved and no shortened bands will be seen on the gel.

**Figure 2:**
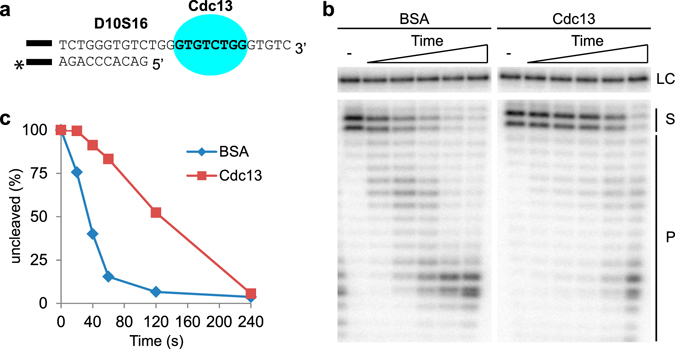
Cdc13 protects the telomere 5′ end when bound 3 nt away from the ds-ss junction. (**a**) D10S16 contains 10 bp of telomere dsDNA and a 16 nt ssDNA 3′ overhang. The Cdc13 MBS (bold text) is located 3 nt from the ds-ss junction. The black bar represents the 14 bp guide sequence (5′-GTCACACGTCACAC-3′) used for ensuring proper annealing. “*” Indicates the radioactive label at the 3′ end of the C-strand, used for detecting the substrate and its degradation products. (**b**) Sequencing gel with the 5′DEPA reaction products. D10S16 was either pre-bound by Cdc13 or incubated with non-DNA binding BSA protein. An aliquot was taken out before addition of λ-exonuclease (-), then λ-exonuclease was added and aliquots of the reactions were stopped at different time points (20; 40; 60; 120; 240 s). (**c**) Graph showing the quantification of the gel shown in (**b**). The amount of uncleaved substrate (S) relative the reaction start point was calculated by measuring the volume of the upper two uncleaved substrate bands normalized to the volume of the loading control band (LC). Reaction products are denoted next to the gel (P). The uncropped gel is presented in Supplementary Fig. S2.

DEPA was performed using a substrate containing 10 bp of telomere dsDNA and a 16 nt telomere ssDNA 3′ overhang. This substrate was named D10S16, and the naming convention D*x*S*y* was used throughout this work to denote the number of *x* bp telomere dsDNA and *y* nt telomere ssDNA in each substrate. D10S16 was either pre-incubated with Cdc13 protein or BSA protein as a negative control, before the addition of the λ-exonuclease (Fig. 2a). Cdc13 binds with high sequence specificity to telomere ssDNA through an 8 nt minimal binding site (MBS) located 3 nt away from the ds-ss junction of D10S16. Electrophoretic Mobility Shift Assay (EMSA) confirmed that Cdc13 binds D10S16 with high affinity under the conditions used for DEPA (Supplementary Fig. S1). When pre-incubated with BSA, λ-exonuclease gradually degrades the C- strand with increasing incubation time (left panel Fig. 2b). However, when the 3′ overhang is bound by Cdc13 the rate of C- strand degradation is strongly reduced (right panel Fig. 2b). The uncleaved substrate is visible as double bands (lane 1 and 7 in Fig. 2b), as a result of contaminating 2′deoxy-ATP being present in the (α-^32^P-) 3′deoxy-ATP used for labelling. Quantitative analyses of the percentage (%) of the substrate that remains uncleaved at increasing incubation times revealed that Cdc13 gives a marked protection against λ-exonuclease compared to BSA control (compare the difference on the y-axis and the slope of the two lines in Fig. 2c). This clearly shows that *N. castellii* Cdc13 can protect the C- strand from 5′-3′ exonucleolytic degradation when bound 3 nt away from the ds-ss junction on the telomere 3′ overhang. This is in line with a previous report showing that *S. cerevisae* Cdc13 can protect a telomere mimicking substrate from degradation by λ-exonuclease^26^.

### Cdc13 protects the telomere 5′ end when bound at various distances from the ds-ss junction

**Figure 3:**
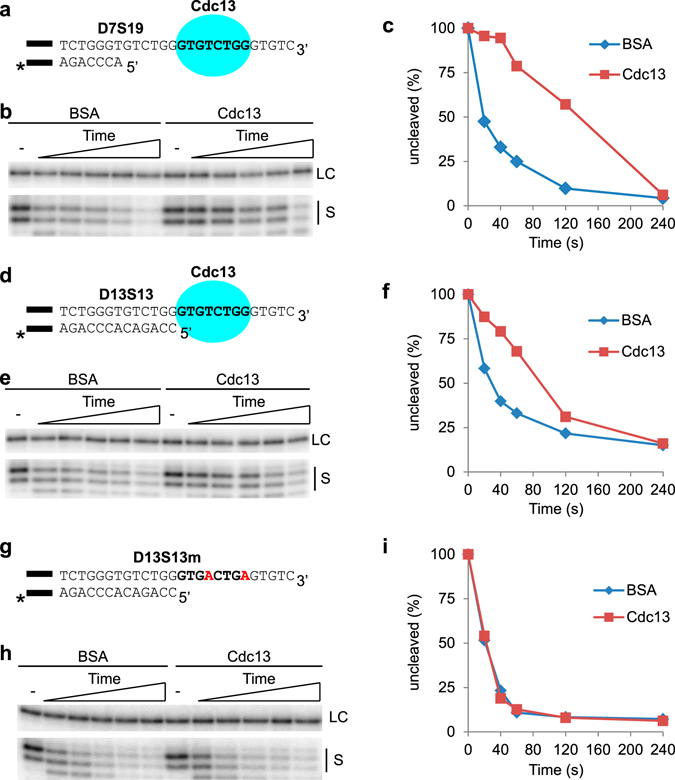
Cdc13 protects the 5′ end when positioned both further away and closer to the ds-ss junction, but not when unable to bind the 3′ overhang. (**a,d,g**) The substrates used for DEPA experiments. The black bar indicates the guide sequence, and “*” the radioactive label (as described in Fig. 2 legend). (**a**) D7S19 contains 7 bp of telomere dsDNA and a 19 nt ssDNA telomere 3′ overhang. The Cdc13 MBS (bold text) is located 6 nt from the ds-ss junction. (**d**) D13S13 telomeric part contains 13 bp dsDNA and a 13 nt 3′ overhang. The Cdc13 MBS (bold text) is located adjacent to the ds-ss junction. (**g**) D13S13m contains 13 bp of telomere dsDNA and a 13 nt 3′ overhang with mutations in the Cdc13 MBS (shown in red). (**b,e,h**) Sequencing gels of the DEPAs using D7S19 (**b**), D13S13 (**e**) and D13S13m (**h**). The loading control (LC) and uncleaved substrate (S) from the Cdc13 containing and BSA control reactions at 0; 20; 40; 60; 120; 240 s are indicated. (**c,f,i**) Quantification of the DEPA gels shown in (**b**), (**e**) and (**h**) respectively. Quantifications were made as described in Fig. 2 and in material and methods section. Full-size gels showing all reaction products are shown in Supplementary Fig. S2.

Depending on the telomere DNA 5′ end permutation, Cdc13 will be bound at different distances from the ds-ss junction, which could potentially affect its ability to perform 5′ end protection. We wanted to investigate whether the distance between the Cdc13 minimal binding site (MBS) and the ds-ss junction will affect the 5′ end protection. To this end, we performed DEPA using two different model telomere ends; D7S19 which contains a Cdc13 binding site 6 nt away from the ds-ss junction (Fig. 3a and Supplementary Fig. S1), and D13S13 which contains a Cdc13 binding site immediately adjacent to the ds-ss junction (Fig. 3d and Supplementary Fig. S1). Both of these assays showed a far greater proportion of the full length substrates remaining for a longer duration post λ-exonuclease addition in the presence of Cdc13 than in the presence of BSA (Fig. 3b,c,e,f). Thus, it can be concluded that Cdc13 protects from 5′ exonucleolytic degradation both when bound 6 nt away from the ds-ss junction and when bound immediately adjacent to the ds-ss junction.

To confirm that the 5′ end protection mediated by Cdc13 is indeed dependent on its binding to telomere ssDNA, we performed control experiments utilizing a variant of D13S13 that contains two mutations in the Cdc13 minimal binding site (D13S13m, Fig. 3g). EMSA showed that these mutations completely abolished Cdc13 binding (Supplementary Fig. S1d), and DEPA performed on D13S13m showed indistinguishable rates of exonucleolytic degradation in the presence of Cdc13 compared to BSA (Fig. 3h,i). These results confirm that Cdc13 needs to be bound to the 3′ overhang to mediate 5′ end protection.

Hence it can be concluded that Cdc13 bound to telomere 3′ overhangs can mediate 5′ end protection from the λ-exonuclease at several different 5′ end permutations, both when bound immediately adjacent to and further away from the ds-ss junction, indicating that Cdc13 should be able to protect most conceivable 5′ end permutations that may exist *in vivo*.

### Rap1 protects the telomere 5′ end from exonucleolytic degradation

Our results show that Cdc13 can protect most conceivable 5′ end permutations *in vitro*, yet Cdc13 is not required for telomere end protection during G1 phase of the cell cycle *in vivo* ^27^. As the telomere 3′ overhang length varies during the cell cycle in *N. castellii*, it may sometimes be too short to allow for Cdc13 binding^38^. A prime candidate for protecting the telomere under such circumstances is Rap1. *N. castellii* Rap1 binds telomere dsDNA with high specificity, and was recently shown capable also of binding across the ds-ss junction^41, 43^. Therefore, we wanted to investigate the role of *N. castellii* Rap1 in 5′ end protection, and whether it can directly block the action of a 5′-3′ exonuclease.

**Figure 4:**
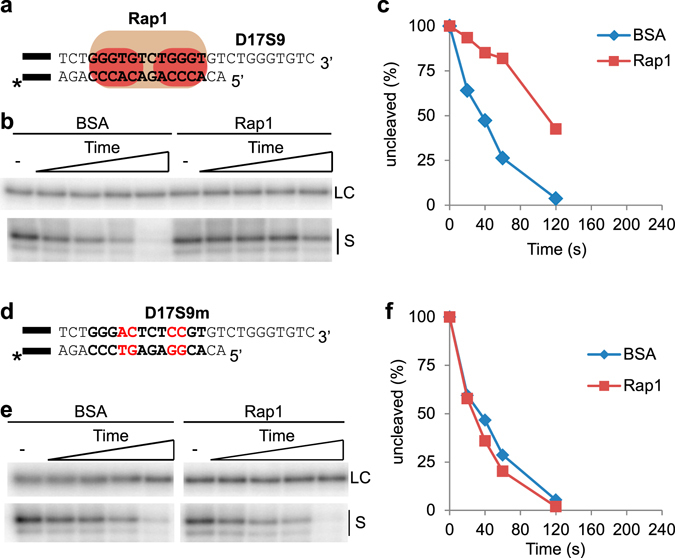
Rap1 protects the 5′ end when bound to dsDNA 2 nt inwards of the ds-ss junction. (**a,d**) The substrates used for DEPA experiments. The black bar indicates the guide sequence, and “*” the radioactive label (as described in Fig. 2 legend). (**a**) The telomere substrate D17S9 contains 17 bp dsDNA and a 9 nt 3′ overhang. The Rap1 MBS (bold text) is located 2 nt inwards of the ds-ss junction. (**d**) Substrate D17S9m contains 17 bp telomere dsDNA with four mutations in the Rap1 MBS (bold letters, mutations in red) and a 9 nt ssDNA 3′ overhang. (**b,e**) DEPA sequencing gels of D17S9 (**b**) and D17S9m (**e**), showing the loading control (LC) and uncleaved substrate (S) from the Rap1 containing and BSA control reactions at 0; 20; 40; 60; 120 s. Full-size gel showing all the reaction products can be found in Supplementary Fig. S4. (**c,f**) Quantification of the gels shown in (**b**) and (**e**) respectively.

To test this, we utilized DEPA with a telomere end containing 17 bp of dsDNA and a 9 nt ssDNA 3′ overhang (D17S9) as substrate. D17S9 contains a Rap1 MBS located 2 nt inbound of the ds-ss junction (Fig. 4a). D17S9 was either pre-bound with Rap1 or incubated with BSA before addition of λ-exonuclease. DEPA revealed clear 5′ end protection mediated by Rap1, as evident by the very slow digestion of the C- strand when pre-bound by Rap1 compared to BSA control (Fig. 4b,c). Interestingly, despite being bound 2 nt inbound of the ds-ss junction, the lack of shortened products shows that the entire length of the C- strand is protected (Supplementary Fig. S4). Further studies showed that Rap1 also protects the entire length of the C- strand when bound to a fully dsDNA Rap1 MBS located 6 nt from the 5′ end (substrate D21S5). This shows that Rap1 can protect the 5′ end even at permutations where it is bound further inward of the ds-ss junction (Supplementary Fig. S5).

To confirm that the 5′ end protection mediated by Rap1 is dependent on DNA-binding, we performed DEPA with a telomere end having mutations in the Rap1 MBS (D17S9m) (Fig. 4d). EMSA confirmed that Rap1 is unable to bind D17S9m (Supplementary Fig. S3), and DEPA revealed that Rap1 is indeed unable to protect D17S9m, since the digestion rate is virtually identical to the BSA control (Fig. 4e,f).

Our results clearly show that *N. castellii* Rap1 bound to telomere dsDNA can inhibit 5′-3′ exonucleolytic activity. Together with reports of *S. cerevisiae* and mammalian Rap1 inhibiting 5′ end resection under certain conditions, it suggests that this might be a conserved function of Rap1^27, 36^.

### The Rap1 DBD is sufficient for 5′ end protection

Rap1 binds telomere dsDNA via a DNA binding domain (DBD) containing two separate Myb-like sub-domains. Having shown that Rap1 protects the 5′ end from exonucleolytic degradation through binding of dsDNA, we hypothesised that the DBD might be sufficient for this Rap1 function.

**Figure 5:**
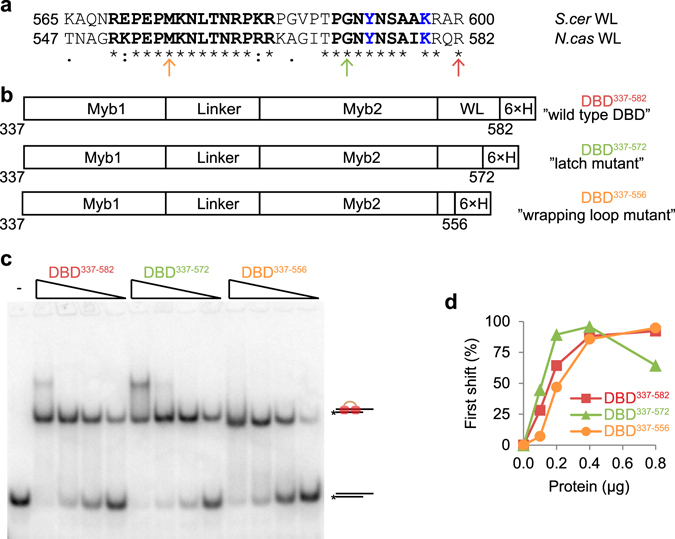
Design of *N. castelli* Rap1 DBD wrapping loop and latch mutants, and analysis of their DNA binding activities. (**a**) Sequence alignment (determined using uniprot CLUSTAL O(1.2.2) multiple sequence alignment tool) of the *S. cer* and *N. cas* wrapping loop. Amino acids of the *S. cer* sequence known to take part in DNA-interactions are shown in bold black text and amino acids interacting with the Myb1 domain are shown in blue. “*” Identical, “**:**” strongly similar, and “.” weakly similar amino acids. The numbers of the first and last amino acids in the wrapping loop are indicated. The last amino acid of each DBD variant is indicated by arrows; orange for “wrapping loop mutant” DBD^337–556^, green for “latch mutant” DBD^337–572^, red for “wild type” DBD^337–582^. (**b**) Schematic of the generated *N. cas* DBD deletion variants, containing two Myb-like domains (Myb1 and Myb2), separated by a linker region, varying lengths of the wrapping loop (WL), and a poly-histidine affinity tag (6ˣH). (**c**) EMSA with D17S9, using, 0.8; 0.4; 0.2; 0.1 μg of the indicated DBD. “-’’ no protein added (**d**) Quantification showing the relative amount of probe (in %) found in the lower shifted band in each lane of the gel in (**d**).

**Figure 6:**
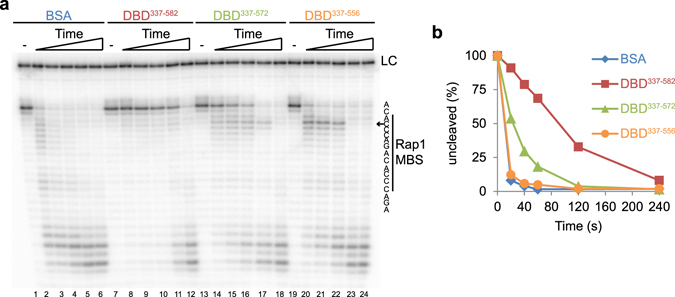
The Rap1 DBD is sufficient and the wrapping loop is essential for mediating 5′ end protection. (**a**) DEPA on D17S9 was performed after pre-incubation with BSA (0.8 μg), Rap1 DBD^337–582^ (0.8 μg), DBD^337–572^ (0.4 μg) or DBD^337–582^ (0.8 μg), for 0; 20; 40; 60; 120; 240 s. The nucleotide sequence representing each band and its position relative to the Rap1 MBS is shown to the right. The arrow points out the position of the stalling product. LC indicates the loading control. Lane number is below the gel. (**b**) The results shown in (**a**) were quantified and the % of uncleaved full length substrate at each time point was calculated.

To test this, a region comprising the entire DBD of *N. castellii* Rap1 was cloned (aa 337–582), expressed as 6x-His tagged fusion protein in *E. coli*, and purified using Ni-NTA affinity chromatography (Fig. 5b Supplementary Fig. S6). Intriguingly, when purified recombinant DBD^337–582^ protein was used for DEPA assays with the D17S9 substrate, it was found to fully protect the 5′ end of the C-strand (Fig. 6a lane 1–12). This was also observed for the full length protein on the same substrate (Fig. 4b). Hence, we conclude that the Rap1 DBD is indeed sufficient for mediating 5′ end protection.

### The Rap1 DBD wrapping loop is required for 5′ end protection

Structural studies of *S. cerevisiae* Rap1 recently revealed that a specific structure, termed the wrapping loop, extends from the C-terminal Myb sub-domain^31^. The wrapping loop folds back and interacts with the major groove of the DNA and forms a latch by interacting with amino acids from the N-terminal Myb sub-domain^31^. Disruption of the latch region or the entire wrapping loop alters the DNA binding properties of the DBD, promoting higher order stoichiometry low affinity binding^44^. We wanted to investigate if the proper high affinity attachment of the Rap1 DBD mediated by the wrapping loop is required for 5′ end protection. To this end, we generated two *N. castellii* DBD mutants, removing the entire wrapping loop or the latch region, respectively. *N. castellii* Rap1 shows high sequence identity to *S. cerevisiae* Rap1 in domains with attributed functions (Supplementary Fig. S6)^41^. Notably, the wrapping loop amino acid residues shown to be involved in DNA-interactions and interactions with the N-terminal Myb domain in *S. cerevisiae* Rap1 show near complete sequence identity in *N. castellii* (Fig. 5a)^31^. We used the information from the structural studies of *S. cerevisiae* Rap1 to generate two DBD mutants that both contain the Myb-like sub-domains of the WT Rap1 DBD, while having a C-terminal truncation that either exclude the latch region of the wrapping loop (DBD^337–572^), or the entire wrapping loop (DBD^337–556^) (Fig. 5b). The proteins were expressed as 6x-His tagged fusion proteins in *E. coli* and purified using Ni-NTA affinity chromatography. Comparison of the DNA binding activities by EMSA of the two Rap1 DBD mutants and the WT Rap1 DBD showed no major differences in affinities for telomere dsDNA on substrate D17S9 (Fig. 5c,d). Similar to what was previously reported for *S. cerevisiae* Rap1^44^, we detected a super-shift at high protein concentrations, which presumably contains two DBD units per DNA substrate.

Next, we used DEPA to compare the ability of each Rap1 DBD variant to protect the 5′ end against exonucleolytic degradation. We used conditions giving 1:1 protein:DNA complex formation as determined by EMSA. When using the D17S9 substrate, which has a Rap1 MBS situated 2 nt inward of the ds-ss junction, DEPA revealed a prominent protection by the wild type DBD^337–582^ as compared to the BSA control (Fig. 6a lanes 1–12 & Fig. 6b). In contrast, the protection was reduced for the latch mutant DBD^337–572^ and completely abolished for the wrapping loop mutant DBD^337–556^ (Fig. 6a lanes 13–24 & Fig. 6b). Intriguingly, while protection of the full length substrates is severely compromised in the DBD^337–556^ wrapping loop mutant, it does however cause stalling of the λ-exonuclease at the fourth nt from the 5′ end, which corresponds to the second nt of the Rap1 MBS (Fig. 6a lanes 20–22). The same stalling is observed for the latch mutant DBD^337–572^, albeit not as marked (Fig. 6a lanes 14–17). Such stalling is not observed in the BSA control, the WT DBD^337–582^, or the full length Rap1 (Fig. 6 & Supplementary Fig. S4). The stalling of the λ-exonuclease suggests altered DNA binding modes of the DBD mutants, allowing the exonuclease to access the 5′ end, then stalling it when it reaches a point where the DBD is more firmly attached.

In conclusion our results show that the wrapping loop region of the Rap1 DBD is essential for its ability to prevent 5′-3′ exonucleolytic degradation by λ-exonuclease.

### 5′ end protection by Rap1 bound to the ds-ss junction is dependent on the length of ssDNA in its minimal binding site

**Figure 7:**
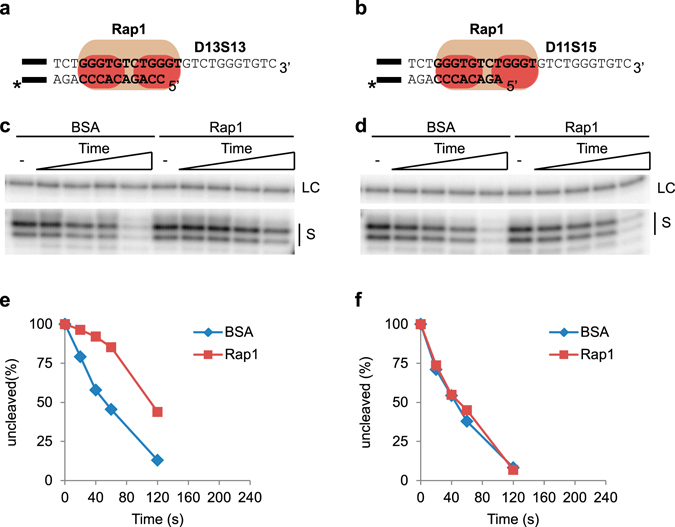
Rap1 protects the 5′ end when its binding site contains 2 nt but not 4 nt of ssDNA. (**a**) Telomere part of D13S13 contains 13 bp dsDNA and a 13 nt 3′ overhang. The Rap1 MBS (bold text) is located across the ds-ss junction and contains 2 nt of ssDNA. (**b**) D11S15 telomere part has 11 bp dsDNA and a 15 nt 3′ overhang. The Rap1 MBS locates across the ds-ss junction and contains 4 nt of ssDNA. The black bar indicates the guide sequence, and “*” the radioactive label (as described in Fig. 2 legend). (**c, d**) DEPA gels showing the results from D13S13 (**c**) and D11S15 (**d**). Both (**c**) and (**d**) show the loading control (LC) and uncleaved substrate (S) from the Rap1 containing and BSA control reactions at 0; 20; 40; 60; 120 s. Uncropped gels can be found in Supplementary Fig. S4. (**e,f**) Quantification of gels shown in (**c**) and (**d**) respectively.

Rap1 is known to bind DNA also in other modes than its high affinity interaction with telomere dsDNA. Recently *N. castellii* Rap1 was shown to bind across the ds-ss junction and interact with the ssDNA 3′ overhang^43^, and subsequently human Rap1 was also shown to bind ds-ss DNA junctions^45^. While conservation across species suggests this binding mode is important, its function remains unknown. When utilizing this binding mode, some 5′ end permutations will lead to Rap1 competing with Cdc13 for binding the ssDNA immediately adjacent to the ds-ss junction^43^. One such permutation is found in substrate D13S13, where Rap1 binds across the ds-ss junction incorporating two nt of ssDNA in its MBS, while Cdc13 binds right next to the ds-ss junction incorporating the same two nt of ssDNA in its MBS (Fig. 3d & Fig. 7a)^43^. However, when Cdc13 binds further away from the ds-ss junction it does not compete with Rap1 bound across the ds-ss junction^43^. Hence, depending on the ability of Rap1 to protect the telomere when bound across the ds-ss junction, the competition between Cdc13 and Rap1 binding might regulate 5′ end resection by 5′-3′ exonucleases.

**Figure 8:**
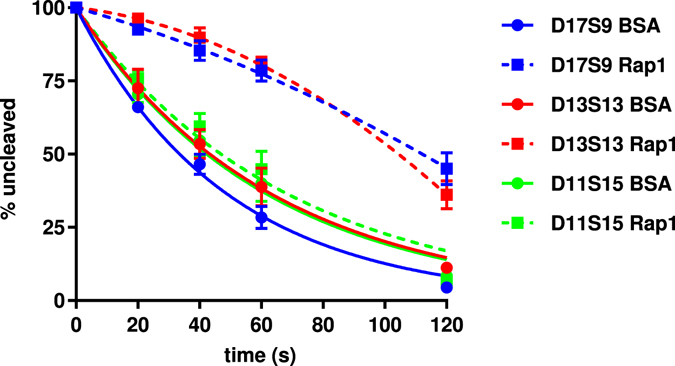
Graph summarizing the results from DEPA experiments assessing Rap1 5′ end protection of the indicated telomere substrates. Each point is the average and error bars ± SEM of three experiments.

To study this possibility further, we employed DEPA to compare the ability of Rap1 to protect telomere ends with different 5′ end permutations. We used substrate D13S13 where the Rap1 MBS located across the ds-ss junction contains 2 nt ssDNA (Fig. 7a), and D11S15 where the Rap1 MBS located across the ds-ss junction contains 4 nt ssDNA (Fig. 7b). DEPA revealed that Rap1 bound to D13S13 gives clear 5′ end protection compared to the BSA control (Fig. 7c,e). In contrast, Rap1 bound to D11S15 gives no added protection, as shown by the virtually identical rate of digestion compared to the BSA control (Fig. 7d,f, Supplementary Fig. S4). Thus, the additional ssDNA included within the Rap1 MBS reduces the capacity of Rap1 to protect the 5′ end. When comparing the results between DEPAs performed with Rap1 bound to D17S9, D13S13 and D11S15 (i.e. the MBS ending 2 nt inward, 2 nt outward, and 4 nt outward of the ds-ss junction respectively) it is clear that Rap1 can provide protection to the telomere 5′ end as long as its MBS contains ≤2 nt of ssDNA (Fig. 8). The diminished protection of D11S15 might be explained by an altered DNA binding mode of Rap1 for this substrate, as indicated by EMSA showing that Rap1 has a somewhat lower affinity for D11S15 than for D17S9 and D13S13 (>90% binding for D17S9 and D13S13 compared to about 66% for D11S15 at the same Rap1 concentration, Supplementary Fig. S7).

Together these results show that the ability of Rap1 to protect the 5′ end varies depending on the amount of ssDNA in its binding site, correlating with its lowered affinity for substrates containing additional bases of ssDNA in the MBS.

## Discussion

We have used an *in vitro* λ-exonuclease based assay to study how the binding of Cdc13 and Rap1 to *N. castellii* telomere ends mediate protection from 5′ exonucleolytic degradation. We find that both Cdc13 and Rap1 can protect the 5′ end, both when bound close to and further away from the ds-ss junction. This may be important for 5′ end protection *in vivo*, where 3′ overhang length is variable and changes in different phases of the cell cycle. Our results highlight the role of Rap1 in 5′ end protection, and to our knowledge is the first report of an *in vitro* biochemical assay showing that Rap1 can directly inhibit a 5′ exonuclease at telomeres. We find that Rap1 efficiently protects against 5′ exonucleolytic degradation when bound to dsDNA, and that the Rap1 DBD is sufficient for mediating this 5′ end protection (Figs 4 & 6). We found that a region of the *N. castellii* Rap1 DBD, previously identified as a wrapping loop and latch in structural studies of the *S. cerevisiae* Rap1, is essential for the ability of Rap1 to protect against 5′ exonucleolytic degradation (Figs 5 & 6)^31^.

**Figure 9:**
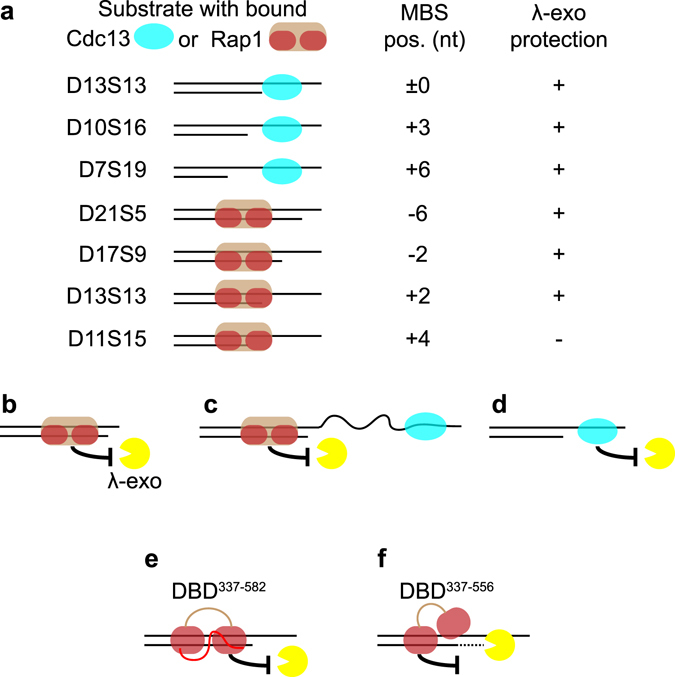
Schematic figure summarizing the results of this work and how it is proposed to relate to different *in vivo* situations. (**a**) Shows the different substrate tested with Cdc13 or Rap1 pre-bound at their respective MBS at various distances relative the ds-ss junction. “ + ” indicates protection, while “−’’ indicates no protection. (**b**) Protection by Rap1 when the 3′ overhang is very short and unable to accommodate Cdc13 binding. (**c**) Protection by Rap1 in a hypothetical situation where Cdc13 is bound very far away from the ds-ss junction (longer than tested here). (**d**) Protection may be provided by Cdc13 alone when the 3′ overhang accommodates its binding. (**e**) The wild type Rap1 DBD^337–582^ is firmly attached to its MBS, and fully protects the 5′ end from degradation by λ-exonuclease. (**f**) The Rap1 wrapping loop mutant DBD^337–556^ is only partly attached to the MBS, leaving the 5′ end accessible to λ-exonuclease, which cleaves off the first 3 nt of DNA before being halted at a site where the mutant DBD is more firmly attached.

Taken together our DNA end protection assays (DEPAs) studying Cdc13 and Rap1 bound at different telomere ds-ss junctions suggest that the two proteins should be able to protect the 5′ telomere end from exonucleolytic degradation at all conceivable 5′ permutations and different 3′ overhang lengths (summarized in Fig. 9). Notably in this regard, Rap1 is able to provide 5′ end protection in situations where the overhang is too short to accommodate Cdc13 binding. This might explain the importance of Rap1 in preventing 5′ end resection in quiescent *S. cerevisiae* cells^27^. Another possible scenario in *N. castellii*, where a subset of telomere 3′ overhangs are up to 200 nt long, is that Cdc13 may be bound at a distance too far away from the ds-ss junction to provide any physical barrier to 5′ exonucleases. In such cases, Rap1 should also be able to provide sufficient 5′ end protection. In other words, Rap1 and Cdc13 have complementary roles in the 5′ end protection. As long as either Rap1 or Cdc13 is attached in proximity to telomere ends they can provide a physical barrier that inhibits 5′ exonucleolytic degradation. This suggests that a temporary removal of these proteins from the telomere end would be necessary for enabling 5′ resection, which in turn is required for creating a suitable substrate for telomere lengthening by telomerase.

Both in the ciliate Euplotes and in human cells, there is a strong preference for a specific telomere 5′ end permutation, which suggests that the mechanism generating the 3′ overhang is highly sequence specific and that it may be conserved in all eukaryotes^10, 46^. Since we found that Rap1 and Cdc13 can protect virtually all conceivable 5′ end permutations, it implies that generation of any possibly preferred 5′ end permutation in *N. castellii* would not result from Rap1 or Cdc13 being able to better protect one particular 5′ end-sequence from exonucleases. Instead, such a preference could be governed by the last RNA priming event in C-strand synthesis, as has previously been proposed for human cells^47^.

Our DEPA experiments showed that the WT Rap1 DBD^337–582^ could fully protect the 5′ telomere end, while a truncated Rap1 DBD variant lacking the entire wrapping loop (DBD^337–556^) showed prominent stalling of the λ-exonuclease at the second nt of the Rap1 MBS (Fig. 6a lane 20–22). This strongly suggests that the binding of the Rap1 DBD^337–556^ wrapping loop mutant to dsDNA is altered. Recently *S. cerevisiae* Rap1 wrapping loop mutants were shown to form high stoichiometry low affinity complexes with dsDNA to a greater extent than the WT Rap1 DBD did^44^. This was proposed to result from two mutant Rap1 DBDs binding one half-site each through attachment of only one Myb-subdomain of each DBD^44^. Our DEPAs were run using protein concentrations generating a single shift in EMSA, which suggest that even when bound as a 1:1 protein:DNA complex, the binding of the wrapping loop mutant is altered (Figs 5c & 6). Our results support a model in which one of the Rap1 Myb-subdomains is not properly attached to its half-site in the wrapping loop mutants. If the Myb-subdomain at the 5′-most half site is loosely attached it could allow access of λ-exonuclease to the 5′ end, while the more firmly attached Myb-subdomain might still be sufficient to stall the exonuclease as it approaches the second half-site (Fig. 9e,f). Hopefully, the insights gained from these *in vitro* studies can be used in future *in vivo* studies aimed at elucidating the role of Rap1 in regulating 5′ end resection, and to separate this function from other essential functions of Rap1.

Rap1 was recently found to also bind across the ds-ss junction and thus to compete with Cdc13 for binding at particular 5′ end permutations, leading us to investigate how this alternative Rap1 binding mode might affect 5′ end protection^43^. We found that Rap1 protects the 5′ end from exonucleolytic degradation when its MBS contains two nt of ssDNA (substrate D13S13, Fig. 7), but not when it contains four nt of ssDNA (substrate D11S15, Fig. 7). This might be explained by Rap1 having lower affinity for the latter substrate. Rap1 and Cdc13 were previously shown to compete for binding of the D13S13 substrate^43^. On the other hand, our present results show that both Rap1 and Cdc13 can protect this junction individually, which indicates that regardless of which protein wins this competition *in vivo* the junction can be protected from 5′ exonucleolytic degradation by either of the proteins. In the situation where Rap1 binds four nt of ssDNA (D11S15), Cdc13 would be expected to bind the 3′ overhang and protect the 5′ end. However, even in a situation where Cdc13 is not present at such a ds-ss junction, Rap1 is expected to bind inward of the junction at its higher affinity binding site consisting entirely of dsDNA. Our results indicate that Rap1 should be able to protect the 5′ end also from this position since it could protect at a 6 nt distance to the ds-ss junction (substrate D21S5, Supplementary Fig. S5). The D21S5 substrate allows for an alternative binding site of Rap1 across the ds-ss junction, including 2 nt of ssDNA. However, previous binding studies have suggested that Rap1 primarily binds the fully dsDNA binding site on this substrate, thus verifying that the protection we see in our DEPAs is indeed mediated by Rap1 being bound to the dsDNA binding site (see Fig. S4 of ref. 43). Hence, our data indicate that Rap1 can protect the 5′ end both when bound at a distance inward of the ds-ss junction, and when bound across the ds-ss junction, incorporating up to two nt of ssDNA (Fig. 8).

The experiments presented here show how telomere binding proteins provide a physical barrier to a 5′ exonuclease at various ds-ss junctions *in vitro* by utilizing that, in contrast to *S. cerevisiae*, *N. castellii* has homogeneous telomere repeats and well-defined Cdc13 and Rap1 binding sites^40–42^. *S. cerevisiae* Cdc13 was recently reported to protect the 5′ end of a telomere mimetic substrate from degradation by λ-exonuclease^26^. Our results confirm that this is the case also for *N. castellii* Cdc13, and further show that Cdc13 can protect the 5′ end both when bound close to and further away from the ds-ss junction. Thus, our results suggest that in budding yeasts with variant telomere repeats, such as *S. cerevisiae*, Cdc13 might protect the 5′ end even if a variant repeat in the 3′ overhang forces it to bind at a distance from the d¡s-ss junction. Our results also indicate that Rap1 may have a more prominent role in protecting the 5′ end of telomeres *in vivo* than previously thought. Intriguingly this has also been suggested in recent reports of the situation *in vivo* in both *S. cerevisiae* and human cells. In *S. cerevisiae*, Cdc13 was attributed the function of preventing accumulation of telomere ssDNA early during the characterization of the *cdc13–1* temperature sensitive mutant, while Rap1 has mainly been associated with a range of other essential aspects of telomere maintenance, and was only recently shown to have a role in preventing accumulation of ssDNA at telomeres in *S. cerevisiae* cells^6, 27, 29^. While Rap1 is the most evolutionarily conserved of the telomere binding proteins found in mammals, its role in mammalian telomere maintenance is not as prominent as in budding yeast, and its precise functions in the Shelterin complex has remained elusive. A recent report does however show a function of the TRF2-Rap1 complex in preventing telomere 5′ end resection and homologous recombination-mediated deletions and fusions in mammalian cells^36^. Furthermore, a study of Rap1^−/−^ Terc^−/−^ mice indicated an important role for Rap1 in protecting telomeres following their shortening due to telomerase deficiency^48^. Rap1^−/−^ Terc^−/−^ mice showed accelerated aging due to telomere shortening in highly proliferative tissues and an increase in telomere fusions similar to those observed upon loss of the TRF2-Rap1 complex^36, 48^. While telomere overhang length in Rap1^−/−^ Terc^−/−^ mice was not reported, the observed phenotype is in line with Rap1 preventing excessive 5′ end resection at short telomeres^48^. Together these reports suggest that Rap1 may have an evolutionarily conserved function in limiting 5′ end resection.

The generation of 3′ overhangs of proper length is important for preventing aberrant DNA damage repair and genomic instability, to allow telomere lengthening by telomerase, and for regulating the rate of telomere shortening in telomerase negative cells^6, 8^. Hence the regulation of 3′ overhang length is of relevance both for understanding the development of cancer, where telomere lengthening and genomic instability are hallmarks of the disease, and for understanding normal aging, where telomere length is known to be of crucial importance for regulating cellular senescence^49–51^. Detailed knowledge about these mechanisms may thus prove valuable in cancer therapies designed to prevent telomere lengthening, or in therapies aimed at prolonging healthy life span by delaying telomere shortening.

## Methods

### Oligonucleotides

Oligonucleotides used in this study (Supplementary Table 1) were custom synthesized at Eurofins genomics (Germany), and the oligos with 5′-phospho modifications were purchased as Hypure (>95% pure). All other oligos were gel purified to >95% purity in house.

### Protein Expression and Purification

As previously reported^40^, *CDC13* was cloned into pGEX-6p-1 vector (GE Healthcare) and was expressed in *E. coli* BL21 (DE3) as N-terminal GST-tagged fusion protein. GST-Cdc13 fusion proteins were affinity purified on GS4B- glutathione resin (GE Healthcare), washed with 1x PBS (pH 7.4) and eluted with 10 mM reduced glutathione in 50 mM Tris-Cl, pH 8.0, following which the GST tag was cleaved off by incubating with PreScission protease (GE Healthcare) in elution buffer at 4 °C overnight. The tag-free Cdc13 fractions were buffer exchanged on PD-10 desalting column (GE Healthcare) into a Cdc13 storage buffer (50 mM NaPO_4_, pH 7.5, 300 mM NaCl, 5 mM DTT, 10% glycerol, modified from ref. 52) and stored at −20 °C until use.

*Ncas-RAP1* was PCR amplified from *N. castellii* genomic DNA (primers: FP- 5′-taattctagagatgtcaagtcctc-3′ and RP-5′-taatctcgagtttaaaccaaatctc-3; XbaI & XhoI sites underlined, respectively) and cloned into pOET1N 6x-His baculovirus transfer vector. Sequence verified recombinant *RAP1* insert with 6x-His tag was sub-cloned (PCR primers: FP-5′ggaagatctgccaccatggtccatcatcaccaccatc3′ and RP-5′cggggtaccttattaaaccaaatctctttccacaaac-3′;BglII& KpnI site underlined) into BamHI/KpnI site of pFastbac1 (Invitrogen).pFastbac1-RAP1 plasmid was used to generate recombinant bacmid using the Bac-to-Bac system (Invitrogen), and transfected into Sf9 cells with FlashFECTIN (OET) according to the manufacturer′s instructions to generate recombinant P3 virus stock. N-terminal 6x-His tagged Rap1 was expressed from Hi5 cells in SFX insect culture medium (HyClone) by infecting with P3 virus stock at multiplicity of infection of 1:10. Cells were grown for 3 days at 100 rpm, 27 °C and were harvested at 4500 × *g* for 30 min at 4 °C. Cell pellets were suspended in 1x Rap1 lysis buffer (20 mM Tris-Cl, pH 8.0, 500 mM NaCl, 1 mM DTT, 10% glycerol, 1 mM Pefablock (Sigma) and 1x complete EDTA-free protease inhibitor (Roche), sonicated (Sonics (Vibracell); Output 60, 50% Pulsed 10 sec ON 10 Sec OFF, 30 pulses on ice) and the lysate clarified at 1000 × *g* by centrifugation for 30 min, 4 °C was affinity purified through HisTrap HP Ni-NTA column (GE Healthcare). Rap1 was eluted from Ni-NTA column by a imidazole gradient between 10–500 mM in 20 mM Tris-Cl, pH 8.0, 500 mM NaCl). The peak fractions containing Rap1-FL protein were pooled and buffer exchanged against Rap1 storage buffer (20 mM HEPES, pH 8.0, 150 mM NaCl, 1 mM DTT, 10% glycerol, 1x protease inhibitor cocktail (Roche)) on PD-10 column (GE Healthcare) and stored at −80 °C until use.

Sequence encoding DNA binding domain (DBD) of Rap1 was PCR amplified from *N. castellii* genomic DNA (Primers Rap1DBD-FP:5′-caccatgttaccatctcataataaag-3′ and Rap1DBD-RP:5′-tctctgcctcttaattgcc-3′) and was TOPO cloned into pET101/D-TOPO vector (Invitrogen), to produce a fusion construct with C-terminal 6x-His tag. Rap1-DBD mutants, Rap1-DBD^337**–**572^ and Rap1-DBD^337**–**556^ were created by site-directed mutagenesis (QuickChange II XL kit, Agilent Technologies) using pET101/D-TOPO-Rap1-DBD as the template using del238–247 FP/RP (5′-caggtataacgcctggtaagggcgagctcaattc-3′/5′-gaattgagctcgcccttaccaggcgttatacctg-3′) and del222–247 FP/RP (5′-gaattgagctcgcccttcatgggttctggtttcc-3′/5′-ggaaaccagaacccatgaagggcgagctcaattc-3′), respectively, according to manufacturer′s protocol. All the constructs were sequence verified before transforming into *E. coli* BL21Star (DE3) (Invitrogen) for expression. Expression of Rap1-DBD and DBD mutants were induced from respective recombinant *E. coli* BL21Star (DE3) cells with 0.5 mM IPTG at OD_600_ of 0.5 for 4 h at 30 °C, 200 rpm. The cell pellets were lysed in 1x DBD lysis buffer (50 mM Tris-Cl, pH 7.5, 1.0% Triton-x-100, 1 mM EDTA, 10% glycerol, 500 mM NaCl, 5 mM DTT, 1x complete EDTA free protease Inhibitor (Roche) and 1 mg/ml lysozyme) by sonication (30% Output, 50% pulsed, 20 sec ON 20 sec OFF for 6 cycles) and lysate clarified at 23708x*g*, 30 min by centrifugation and 0.45 μm filtration was loaded on to HisTrap Ni-NTA column (GE Healthcare), washed with wash buffer (50 mM NaPO_4_, pH 7.5, 500 mM NaCl, 20% glycerol, 5 mM DTT and 10 mM imidazole) and eluted with 100 mM to 250 mM imidazole in wash buffer. The fractions containing >90% pure Rap1-DBD or DBD mutants were pooled and buffer exchanged to Hi-salt Rap1-DBD storage buffer (20 mM HEPES, pH 7.6, 500 mM NaCl, 5 mM MgCl_2_, 1 mM DTT, 0.5 mM EDTA and 20% glycerol) on PD-10 column and stored at −80 °C. Before use, small portions were buffer exchanged in to a low-salt Rap1-DBD buffer (20 mM HEPES, pH 7.6, 150 mM NaCl, 5 mM MgCl_2_, 1 mM DTT, and 10% glycerol) on PD-10 columns and stored frozen as aliquots at −20 °C until use.

### Electrophoretic Mobility Shift Assay (EMSA)

DNA binding of telomere binding proteins was assayed using EMSA. Various partially single-stranded telomeric probes mimicking the possible ds-ss junction permutations in *N. castellii* telomere were used in this study (Supplementary Table 1). All the oligonucleotides contain a 14 nucleotides (nt) non-telomeric region which direct the annealing between the forward G-strand and the reverse C-strand. Oligonucleotides were named according to the length of double-stranded (D) and Single-stranded (S) telomeric sequence in the probe. In order to create different ds-ss junctions, C-strands with varying lengths ending in one of the eight possible 5′ permutations of *N. castellii* were used.

The reverse strand was radioactively labelled with chain terminator (α-^32^P-) 3′deoxy-ATP (Perkin Elmer) at the 3′end using terminal deoxynuclotide transferase (TDT) (New England Biolabs) according to manufacturer′s protocol. Radiolabelled reverse strands were annealed with 1.5x excess of unlabelled forward strand in annealing buffer (1 mM Tris-Cl, pH 8.0, 0.1 mM MgCl_2_) by boiling for 2 min and cooling down overnight. The successful creation of partial ds probe was assayed by mobility shift compared to ssDNA reverse strand on 6% EMSA gel.

For the binding assay, 10 fmol probe in presence of 1.5 µg competitor mix (0.5 µg each of sheared *E.coli* DNA (~250 bp), salmon sperm DNA and yeast t-RNA) in 1x λ-exonuclease buffer (New England Biolabs; 67 mM Glycine-KOH, pH 9.4, 2.5 MgCl_2_ and 50 µg/µl BSA) supplemented with 8% glycerol was mixed with varying concentrations of affinity purified Cdc13 (~0.8–4.8 μg), Rap1 (~0.07–7 μg), Rap1-DBD or DBD-mutants (~0.1–1.6 μg), in a total of 15 µl reaction. The reaction was carried out at 25 °C for 15 min and samples were loaded onto 6% polyacrylamide (29:1 acrylamide:bis-acrylamide) gel and run in 1x TBE (89 mM Tris-borate, 2 mM EDTA, pH 8.0), 150 V at 4 °C. The concentration of each of the protein required to produce a saturated 1:1 binding with 10 fmol probe, as determined by EMSA, was used in performing DEPA.

### *In vitro* 5′ DNA End Protection Assay (DEPA)

In order to assay the ability of Cdc13 and Rap1 in providing protection to 5′ ends of the budding yeast telomere, we have developed an *in vitro* DEPA using the *N. castellii* telomere sequence and proteins. In this assay *N. castellii* telomere-mimicking, partially single-stranded probes, having a single minimal binding site (MBS) for Rap1 or Cdc13, were radiolabelled at 3′ end of C–strand and were subjected to digestion by λ-exonucleases in the presence or absence of these proteins. The protection provided to the 5′ end against degradation by 5′exonucleases in the presence of Rap1 or Cdc13 was assessed by resolving the digestion reactions on 10% denaturing polyacrylamide gels and phosphoimaging.

Typical protein amount used for DEPAs: 2.4 μg Cdc13, 0.7 μg Rap1 and 0.4–0.8 μg Rap1-DBD or DBD mutants (as stated in legend of Fig. 6). The amounts of proteins used were determined to give 1:1 saturated binding by EMSA. BSA at similar concentration in respective storage buffers served as non-DNA binding negative control.

For DEPA using λ-exonuclease, a master mix (6.5 reactions) was made, where the respective proteins were pre-bound to 65 fmol probe in 1x λ-exonuclease buffer in presence of competitor mix at 25 °C for 15 min (15.6 μg Cdc13 and/or 3.75 µg Rap1 or 2.6 μg Rap1DBD/DBD mutants was used). In the control reaction corresponding amount of BSA in respective buffer was added instead of proteins. At time ‘0’, 15 μl (10 fmol probe) was taken out from both control and test reaction into 9.5 μl stop solution (50 mM Tris-Cl, pH 8.0, 50 mM EDTA, 1.25% SDS, 5 µg Proteinase K) and incubated at 65 °C for 25 min. To the rest of the master reaction λ-exonuclease was added to a final concentration of 0.06 U/μl and incubated at 37 °C. 15 µl samples were taken out at time points indicated in each figure legend and incubated at 65 °C as indicated above. For DNA precipitation, 263 μl sterile distilled water, 32 μl 3 M sodium acetate, pH 5.2, 20 μg glycogen, 5 fmol radiolabelled 40 nt probe (loading control) and 825 µl ice cold 96% ethanol was added to each reaction tube and left at −20 °C for one hour or overnight. Samples were then centrifuged at 13000 rpm for 30 min and the obtained pellets were dried and suspended in 4 μl loading dye (1 mg xylene cyanol, 10 mM EDTA, 95% formamide), heated at 90^o^C for 2 min and loaded onto 10% denaturing polyacrylamide gel (19:1 acrylamide:bis-acrylamide) containing 7 M urea and resolved in 0.6X TBE at 1800 V for 100 min. In some gels the uncleaved substrate is visible as double bands, as a result of contaminating 2′deoxy-ATP being present in the (α-^32^P-) 3′deoxy-ATP used for labelling (according to the manufacturer, Perkin Elmer).

### Gel image acquisition, quantification and processing

Dried gels were exposed to phosphorimaging screens which were scanned using a Typhoon FLA9500 imager (GE Life Sciences) at 200 μm resolution. Files were saved in.gel* format.

Quantifications were done using the ImageQuant TL 1D v8.1 software (GE Life Sciences) on the raw data files prior to image processing. Lanes were manually selected and band grimaces added where required. Background subtraction was performed using “rolling ball” with radius set to 200. Automatic detection of bands was used (minimum slope: 90, median filter: 4), except in cases where a band was expected and the software failed to detect it, in which cases it was manually selected. Band edges were manually adjusted to include the entire band above background in the lane profile. For DEPAs tables with band volume information were exported to Excel (Microsoft) where the volume of the uncleaved substrate bands was normalized to the loading control band of each lane. % uncleaved substrate was then calculated relative to the 0 time point lane. For EMSAs tables with calculated band % were exported to Excel. Before image processing.gel* files were opened in ImageJ and saved as.tif* files. Contrast was then enhanced in Adobe Photoshop CS6 by adjusting the level setting. Images were then cropped before being imported to Adobe Illustrator CS6 where figures were assembled.

Graphs were assembled in Excel before export to Adobe illustrator. At least two independent experiments were performed for each condition and the displayed gels and quantifications show a representative example. Figure 8a was generated using GraphPad Prism 7 and the fitted curves are “one phase decay” for all except D17S9 Rap1 and D13S13 which are second order polynomial (quadratic) fits.

## Electronic supplementary material

Supplementary information
