# Supplementary material for: Rap1 and Cdc13 have complementary roles in preventing exonucleolytic degradation of telomere 5′ ends
Source: Sci Rep. 2017 Aug 18;7:8729. doi: 10.1038/s41598-017-08663-x (PMC5562816; doi:10.1038/s41598-017-08663-x)
Supplement: Supplementary file 1 — Supplementary information [file 41598_2017_8663_MOESM1_ESM.pdf]

Supplementary Information

Rap1 and Cdc13 have complementary roles in preventing exonucleolytic degradation of telomere 5' ends

Rikard Runnberg, Saishyam Narayanan, Marita Cohn*

Table S1. List of oligonucleotides used in this study written in 5'-3' direction. Purple letters indicate non-telomeric guide sequence used for directing the correct annealing, small letter indicate a mutation and italic indicates 5'-phosphorylation. The description states which substrates that are made using each oligonucleotide (where Dx, Sy, in the substrate name indicates the number of telomere double and single stranded nucleotides, respectively).

G-rich strand sequence (5'-3')	Used for preparing
GTCACACGTCACACTCTGGGTGTCTGGGTGTCTGGGTGTC	All except D17S9m and D13S13m
GTCACACGTCACACTCTGGGacTCTccGTGTCTGGGTGTC	D17S9m
GTCACACGTCACACTCTGGGTGTCTGGGTGaCTGaGTGTC	D13S13m
**C-rich compimentary strand sequence (5'-3')**	**Used for preparing**
*C*CAGACACCCAGACACCCAGAGTGTGACGTGTGAC	D21S5
*A*CACCCAGACACCCAGAGTGTGACGTGTGAC	D17S9
*A*CACggAGAgtCCCAGAGTGTGACGTGTGAC	D17S9m
*C*CAGACACCCAGAGTGTGACGTGTGAC	D13S13 and D13S13m
*A*GACACCCAGAGTGTGACGTGTGAC	D11S15
*G*ACACCCAGAGTGTGACGTGTGAC	D10S16
*A*CCCAGAGTGTGACGTGTGAC	D7S19

Figure S1

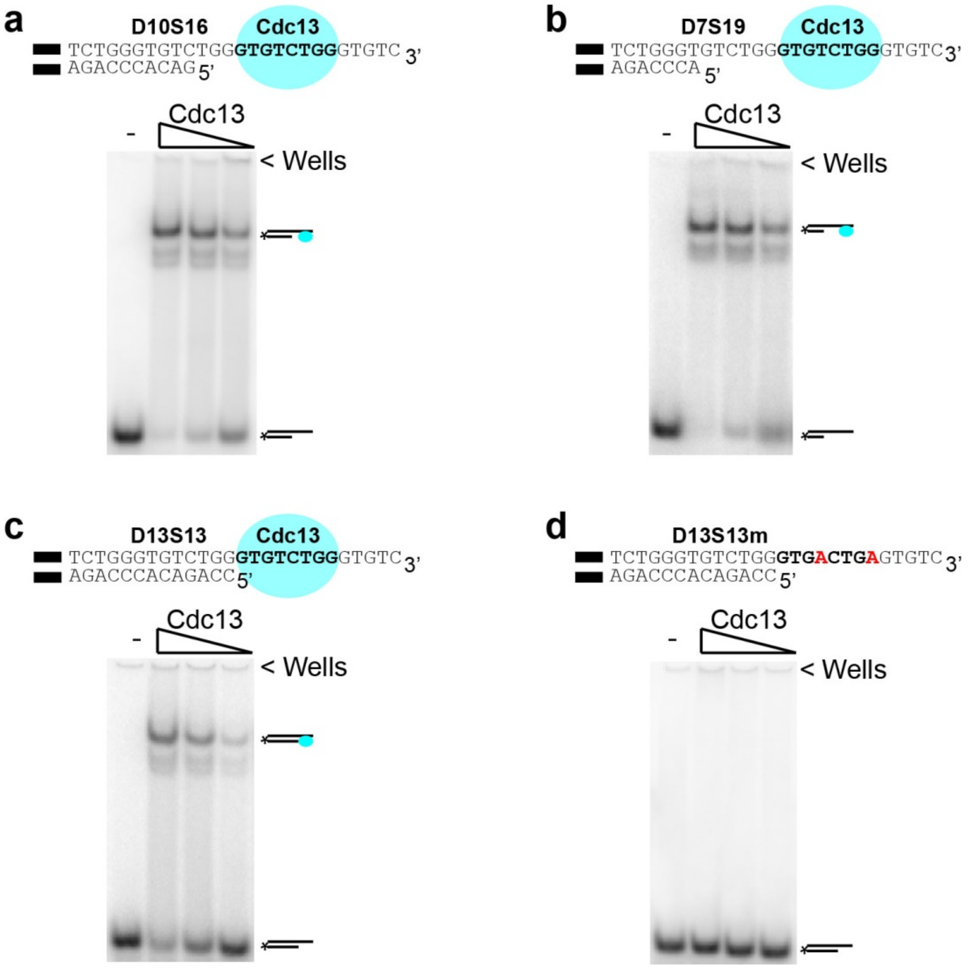

Figure S1. EMSA showing the binding of Cdc13 protein to substrates D10S16 (**a**), D7S19 (**b**), D13S13 (**c**) and D13S13m (**d**). 2x dilution series of Cdc13 was used. Free labelled probe, the major shift containing Cdc13 bound probe, and the wells of each gel are indicated.

Figure S2

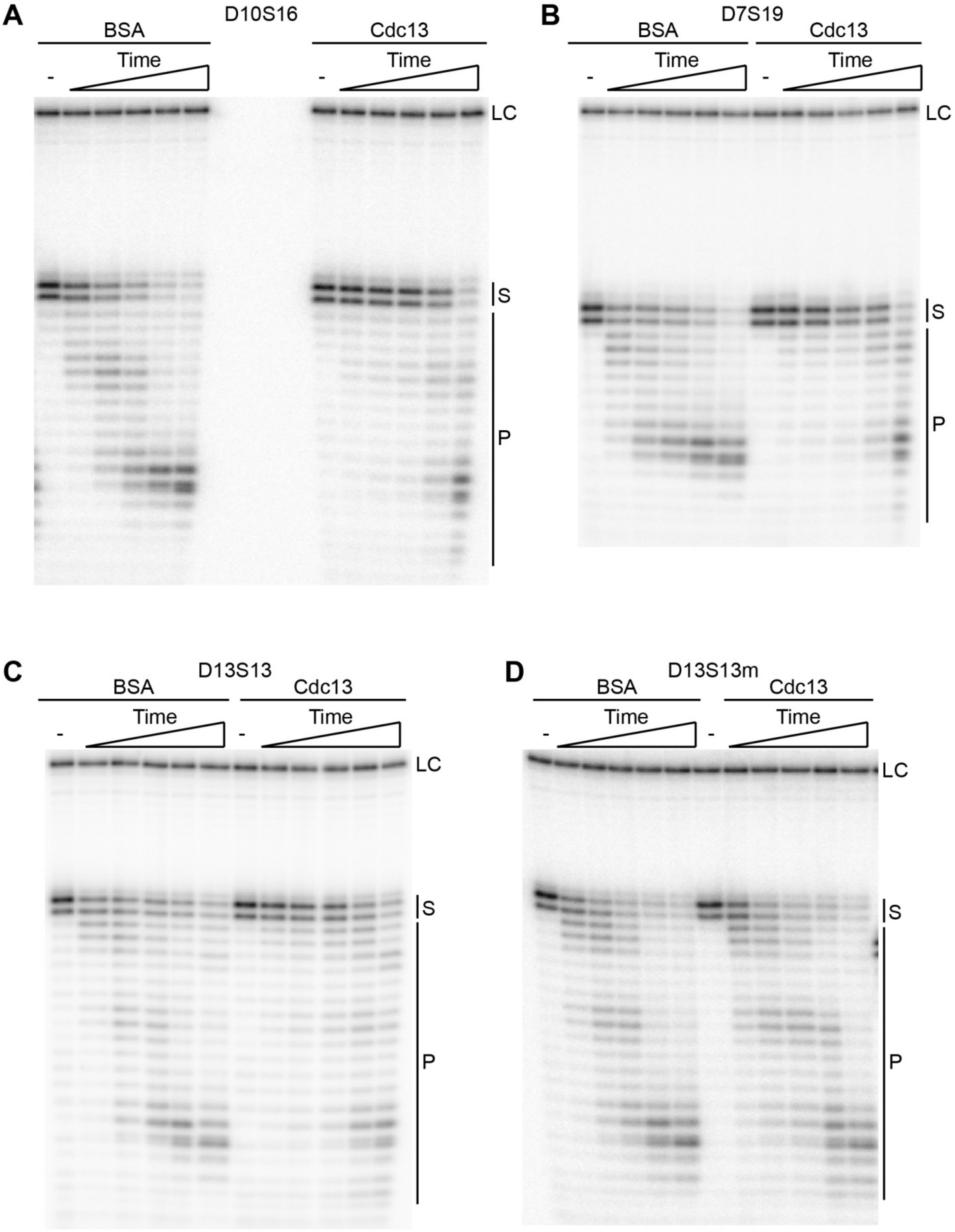

Figure S2. Uncropped gels of 5'DEPAs of D10S16 presented in main manuscript Fig. 2 (**a**), D7S19 presented in main manuscript Fig. 3 (**b**), D13S13 presented in main manuscript Fig. 3 (**b**), and D13S13m presented in main manuscript Fig. 3 (**c**). Position of loading control (LC), uncleaved substrate (S), and degradation products (P) is shown next to each gel.

Figure S3

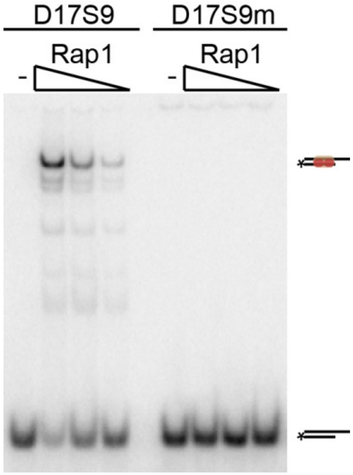

Figure S3. EMSA of Rap1 with D17S9 and D17S9m. Protein added in 2x dilution steps. "-", no protein added.

Figure S4

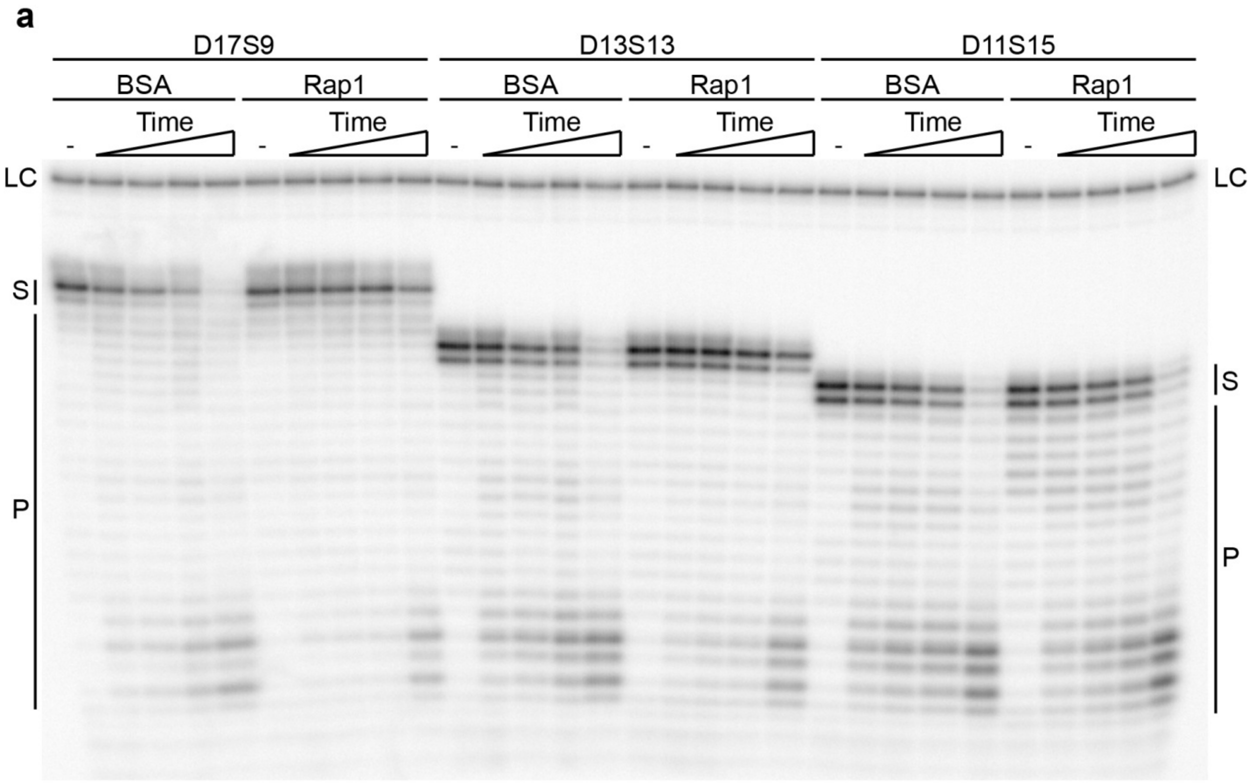

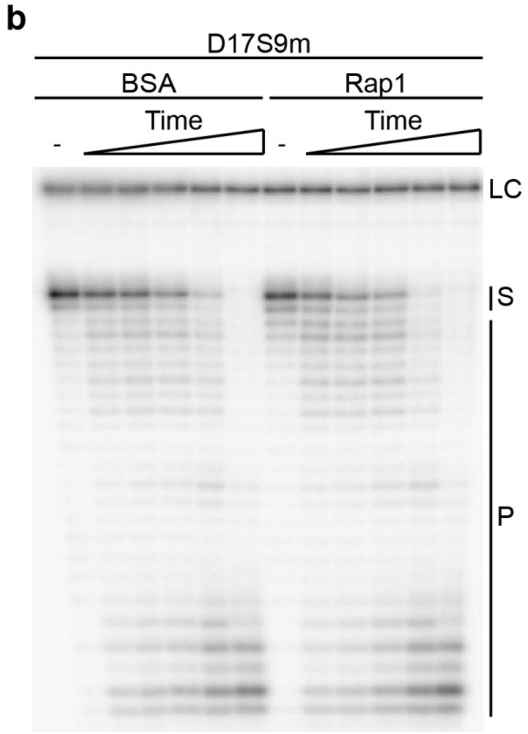

Figure S4. Uncropped gels of the DEPAs shown in main manuscript Fig. 4 and 7. Reactions were stopped at 0; 20; 40; 60; 120 s for (**a**) and 0; 20; 40; 60; 120; 240 s for (**b**). Position of loading control (LC), uncleaved substrate (S), and degradation products (P) is shown next to each gel.

Figure S5

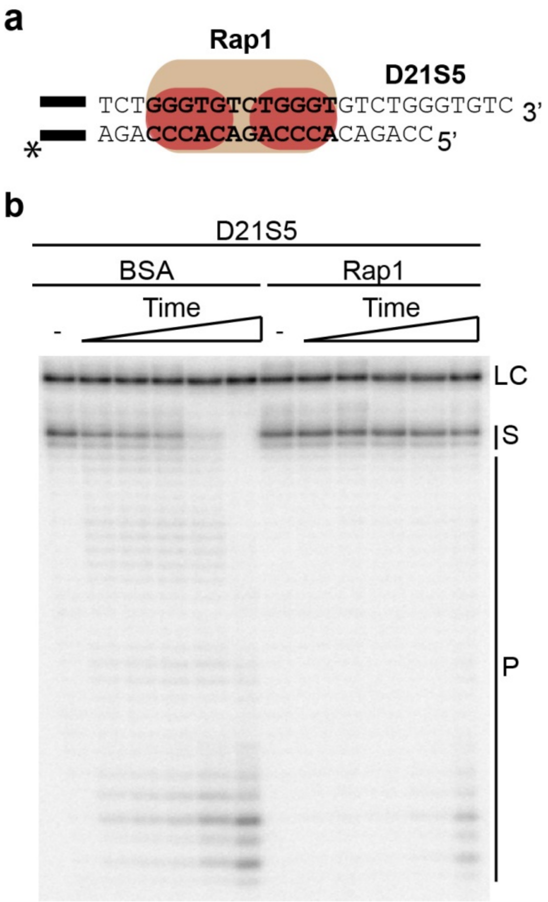

Figure S5. Rap1 protects the 5' end when bound 6 nt inward of the ds-ss-junction. (**a**) Substrate D21S5 contains a fully double stranded Rap1 MBS 6 nt inward from the ds-ss-junction (shown). It also contains a lower affinity binding site (not shown) across the ds-ss junction that incorporates 2 nt ssDNA, which is not the preferred binding site (see discussion for details, and Fig. S4 of Gustafsson, et al. [1]). The black bar represents the 14 bp guide sequence (5'-GTCACACGTCACAC-3') used for ensuring proper annealing. "*" indicates the radioactive label at the 3' end of the C-strand, used for detecting the substrate and its degradation products. (**b**) Sequencing gel showing the DEPA products after pre-incubating D21S5 with either Rap1 or BSA before adding enzyme (-) and at 20; 40; 60; 120; 240 s after the λ-exonuclease was added. Position of loading control (LC), uncleaved substrate (S), and degradation products (P) is shown next to each gel.

Figure S6

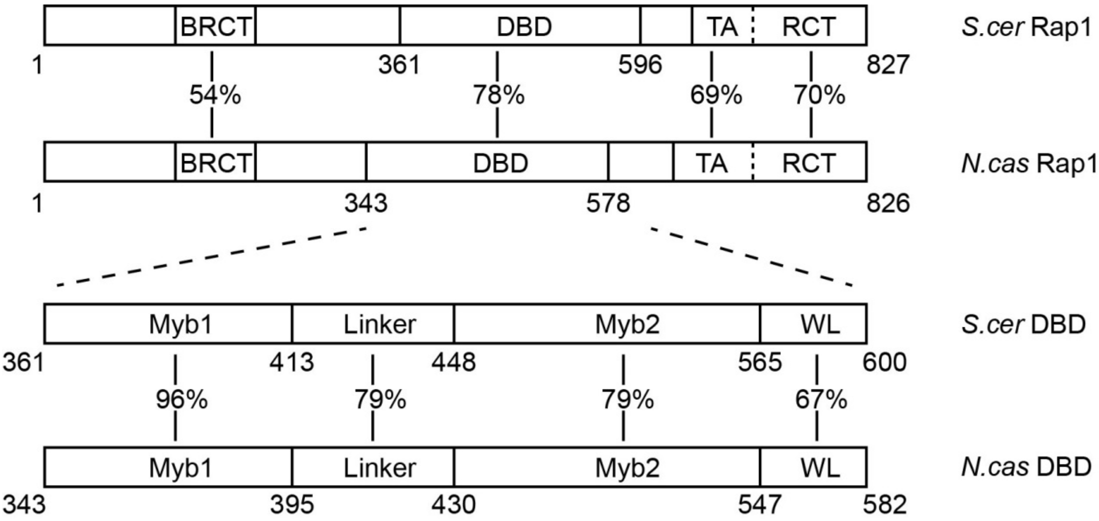

Figure S6. Schematic comparing the Rap1 amino acid sequence from *S. cerevisiae* (*S. cer*) and *N. castellii* (*N. cas*), indicating the sequence identity between specific domains (modified from Wahlin & Cohn[2]) and specific regions of the DBD (determined using uniprot CLUSTAL O 1.2.2 multiple sequence alignment tool). The indicated domains are the BRCA1 C-terminus domain (BRCT), the DNA binding domain (DBD), the transcriptional activator domain (TA), and the Rap1 C-terminal domain (RCT). TA and RCT partially overlap, indicated by a dashed line. The DBD is divided into two Myb-like subdomains (Myb1 and Myb2), separated by a linker region, and contains a wrapping loop (WL) domain.

Figure S7

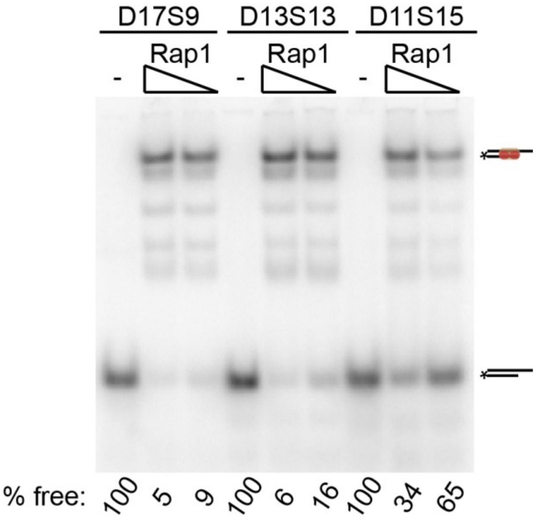

Figure S7. EMSA of Rap1 bound to the indicated substrates. Protein added in 2x dilution steps. "-", no protein added. Relative amounts of labelled probe in each band were quantified. The percentage of free (unbound) probe is shown underneath each lane.

References

1 Gustafsson, C., Rhodin Edsö, J. & Cohn, M. Rap1 Binds Single-stranded DNA at Telomeric Double- and Single-stranded Junctions and Competes with Cdc13 Protein. *Journal of Biological Chemistry* **286**, 45174-45185, doi:10.1074/jbc.M111.300517 (2011).

2 Wahlin, J. & Cohn, M. Analysis of the RAP1 protein binding to homogeneous telomeric repeats in Saccharomyces castellii. *Yeast* **19**, 241-256, doi:10.1002/yea.816 (2002).
